# Asymptomatic Atherosclerosis in Egyptian Rheumatoid Arthritis Patients and Its Relation to Disease Activity

**DOI:** 10.1155/2015/381931

**Published:** 2015-02-08

**Authors:** Rawhya R. Elshereef, Aymen Darwish, Amal Ali, Mohammed Abdel-kadar, Lamiaa Hamdy

**Affiliations:** ^1^Rheumatology and Rehabilitation Department, Minia University, P.O. Box 61519, Minia 61111, Egypt; ^2^Cardiology Department, Minia University, P.O. Box 61519, Minia 61111, Egypt; ^3^Clinical Pathology Department, Minia University, P.O. Box 61519, Minia 61111, Egypt

## Abstract

*Aim*. To detect the frequency of subclinical atherosclerosis in rheumatoid arthritis patients without clinically evident atherosclerosis and to correlate its presence with the disease activity. *Patients and Methods*. Our study includes 112 RA patients (group 1) and 40 healthy controls (group 11). All patients and controls were subjected to full history taking, clinical examination, and laboratory investigations. Carotid intima media wall thickness (IMT) and carotid plaques were measured in both groups by B-mode ultrasonography; also color duplex Doppler ultrasound of the brachial artery was done to detect endothelial function. *Results*. There is atherosclerosis in 31.3% of asymptomatic RA patients compared with only 5% in controls (*P* = 0.003^**^). A significant difference was detected in patients with and without atherosclerosis regarding duration of the disease (*P* = 0.0001^***^) and patient's age (*P* = 0.01^*^). There is highly statistical significant correlation between atherosclerosis and disease activity index. *Conclusion*. The frequency of subclinical atherosclerosis was high in long-term active RA patients.

## 1. Introduction

Rheumatoid arthritis is a chronic inflammatory disease. Cardiovascular events are the most important cause of mortality and morbidity in patients with rheumatoid arthritis [1]. Patients with rheumatoid arthritis (RA) have a two to five times increased risk of developing premature cardiovascular disease that shortens life expectancy by 5–10 years. Thus, in patients with active rheumatoid arthritis, the majority of cardiovascular deaths result from accelerated atherosclerosis [2, 3]. The inflammatory events in RA patients play an important role in acceleration of atherosclerosis process. Many similarities have emerged between the paradigm of inflammation in the pathogenesis of atherosclerosis and the well-established mechanisms of inflammation in the pathogenesis of RA. Hence, the inflammation in RA is not confined to the joints but also present in the vessel wall [2]. Atherosclerosis, previously thought to be a passive disease of lipid accumulation, is now widely acknowledged as a dynamic inflammatory process beginning with endothelial activation, leukocyte recruitment, lipid oxidation, and culminating with plaque destabilization and thrombosis [4]. Subclinical atherosclerosis can be demonstrated by an increased main carotid artery intima media thickness (IMT), a good marker of generalized atherosclerosis. Measurement of carotid artery IMT is a noninvasive, sensitive, cost-effective method to determine subclinical atherosclerosis and to diagnose at-risk patient groups [5, 6].

## 2. Patients and Methods

### 2.1. Patients

#### 2.1.1. Group [I]: Rheumatoid Arthritis Patients

One hundred and twelve patients with RA were included in the present study, 94 women and 18 men; their age ranged from 21 to 49 years and disease duration ranged from six months to fifteen years. Patients were diagnosed according to the American College of Rheumatology (ACR) 1987 revised criteria for the classification of rheumatoid arthritis [7]. They were consecutively recruited from the outpatient clinic of Rheumatology and Rehabilitation Department El-Minia University Hospital. The RA patients were further subdivided according to disease activity into group 1A (patients with moderate and sever RA) and group 1B (mild RA and RA with remission).

*Exclusion Criteria.* Control and RA patients with known atherosclerotic complications such as stroke and MI; those undergoing hemodialysis; patients with peripheral vascular disease, malignancy, or infections; hypertensive and diabetic patients; and smokers were excluded; patients complaining of any cardiac symptoms or signs (chest pain, dyspnea, palpitation, LL edema, etc.) were also excluded.

#### 2.1.2. Group [II]: Control Persons

Forty age and sex matched healthy subjects were recruited as controls for laboratory investigations and duplex ultrasonographic evaluation.

### 2.2. Methods

All patients underwent a complete history taking and clinical examination according to the standard protocol. General and locomotor examinations were done for all patients.

Assessment of disability by the Health Assessment Questionnaire (HAQ) disability index: The Arabic version of disability index of the Stanford Health Assessment Questionnaire was used to assess disability [8] (assessment of pain by visual analog scale: Ranged from no pain degree (o) to worst pain degree (100%); assessment of disease activity by the 28 joint disease activity score (DAS28) [9]). DAS28 includes 10 PIPs, 10MCPs, both wrists, both elbows, both shoulders, and both knees. DAS28 (formula with four variants) is as follows: DAS28 = [0.56√(TEN28) + 0.28√(SW28) + 0.70Ln(ESR) + 0.014(GH)], mild < 3.2, moderate = 3.2–5.1, and severe > 5.1.

#### 2.2.1. Laboratory Investigations

The following was done for all patients and controls: Complete blood count (RBCs, WBCs, and platelets), using an automated cell counter, Sysmex NE; erythrocyte sedimentation rate (ESR) (by Westergren method); rheumatoid factor (RF); latex agglutination slide test for the qualitative and semiquantitative determination of rheumatoid factor in nondiluted serum [10]; liver functions tests, done on dimension ES chemical autoanalyzer: serum aspartate transaminase (AsT), serum alanine transaminase (AlT); lipid profile: total cholesterol (normal up to 130 mg/dL); triglycerides (normal range 50−150 mg/dL); HDL, high density lipoprotein (normal range 0–80 mg/dL); LDL, low density lipoprotein (normal range 80−130 mg/dL); and highly sensitive C-reactive protein (ELIZA).

#### 2.2.2. Imaging Evaluation

Anteroposterior radiographs of both hands and feet were done. Simple Erosion Narrowing Score (SENS) was used for X-ray scoring [11]. It is a simplified method of scoring radiographs based on the Sharp/van der Heijde score [12]. Radiographs were scored by a consultant rheumatologist in random order. The latter was blinded to patients' personal and clinical data at the time of scoring.

#### 2.2.3. Duplex Ultrasonographic Evaluation

Evaluation is done as follows:

1. Duplex ultrasonography of both carotids;
2. ultrasonography to right brachial artery to detect endothelial function.

*(I) Carotid Ultrasound Measurements.* Subclinical atherosclerosis was investigated in RA patients and in control subjects using carotid ultrasound evaluation. Briefly, individuals in the study population were investigated in the supine position, with the head turned away slightly from the sonographer. The common carotid arteries were carefully examined for wall changes in all subjects, obtaining different longitudinal and transverse views with the high-resolution B-mode ultrasound equipment Medison 9900 multibeam 30 UL (Korea) equipped by liner probe (7.5 MHz) with the use of a standardized protocol [13]. A region about 1.5 cm proximal to the carotid bifurcation was identified, and the intima media thickness (IMT) of the far wall was evaluated as the distance between the luminal-intimal interface and the medial-adventitial interface. One transverse and two longitudinal measurements of IMT were obtained from 4 contiguous sites at 2 mm intervals, and the average of the 4 measurements was used for the analysis.

All ultrasound measurements were performed by the same examiner who was unaware of subject characteristics.

This evaluation aims to determine the intima media thickness (IMT) and to detect carotid plaques. The mean IMT (the mean of both right and left sides) was assessed. At the same time the maximum IMT (the highest value either right or left) was also assessed. IMT is considered abnormal if >0.07 cm [14]. Plaques were defined as focal widening relative to adjacent segments, with protrusion into the lumen of calcified or noncalcified material [15].

*(II) Endothelial Function.* To assess endothelial function noninvasively with B-mode ultrasound, conduit vessel endothelium-dependent vasodilatation was induced by reactive hyperemia, while endothelium-independent vasodilatation was induced by administration of sublingual nitroglycerine (glyceryl trinitrate; GTN) as described by Yim and his colleagues [16]. Measurements were made of changes in the diameter of the brachial artery using color duplex Doppler ultrasound. The ultrasound examination was performed in quiet room at temperature between 21°C and 32°C. Subjects rested in a supine position for 15 minutes before examination. AB-mode scan was obtained of the right brachial artery in longitudinal section. A resting measurement was taken (pre-FMD), and a pneumatic cuff was then inflated to a pressure pf 200 mm Hg for 5 minutes; then the diameter of the artery was recorded again 45–60 seconds after deflation (post-FMD). A period of 15 minutes was allowed for recovery before testing for endothelium-independent relaxation. A repeat baseline measurement of the diameter was before a 400 ug dose of sublingual GTN spry was administrated (pre-GTN). The brachial artery diameter was again measured 3-4 minutes after the GTN was given (post-GTN). A single investigator performed all imaging and analysis, blinded to the subject's disease [17].

FMD, GTN, and dilatation were calculated as follows:

(1) $$\begin{array}{c} \text{FMD}=\left\{\frac{\left(\text{Post}\;\text{FMD}-\text{Pre}\;\text{FMD}\right)}{\text{Pre}\;\text{FMD}}\right\}\times 100,\\ \text{GTN}=\left\{\frac{\left(\text{Post}\;\text{GTN}-\text{Pre}\;\text{GTN}\right)}{\text{Pre}\;\text{GTN}}\right\}\times 100,\\ \text{Dilatation}\;\text{ratio}=\frac{\text{FMD}}{\text{GTN}}. \end{array}$$

Data were coded, entered, and analyzed by the statistical package for the Social Sciences (SPSS for windows version 11.0) [18]. Two-tailed tests were used throughout and statistical significance was set at the conventional 0.05 level. The following statistics were carried out. Descriptive statistics: The range, means, and standard deviation were calculated for interval and ordinary variables and frequencies and percentages for categorical variables [19]. Group comparisons: Comparisons were done by two procedures.  ^*^Student's t-test: The independent samples *T* test was used to compare the means of two groups of cases.  ^*^The chi-squared (*χ* ^2^) test: We used the *χ* ^2^ test to test the significance of the differences between the two groups in categorical variables. Correlations: The bivariate correlations procedure computes Pearson's correlation coefficient with its significance levels. Pearson's correlation coefficient is a measure of linear association.

## 3. Results

This study was carried out on 112 RA patients (group 1) and 40 age and sex matched healthy controls (group 11). Patients were classified by DAS28 scores into the following: patients in remission (defined as DAS28 score <2.4) was observed in six out of 112 (5.4%) cases; low disease activity (<3.2) was found in 14 (12.5%); moderate activity (3.2–5.1) in 28 (25%); and high disease activity (>5.1) in 64 (57.1%) RA patients.

### 3.1. Descriptive Study

The demographic data of the RA patients and controls are shown in Table 1.

### 3.2. Patient Characteristics

#### 3.2.1. General Clinical Features of RA Patients

The mean disease duration was 6.03 ± 3.9 years (ranging from 6 months to 15 years) and 19.64% of our patients were of recent onset (<2 years). The DAS28 ranged between 1.2 and 7.81 with a mean of 5.21 ± 1.68. The visual analog scale of pain ranged between 20 and 80 with a mean of 55.71 ± 5.135, while the Health Assessment Questionnaire-Disability Index ranged between 0 and 1.77 with a mean of 0.742 ± 0.501. The body mass index ranged between 17.7 and 42.25 with a mean of 26.02 ± 6.42; also the waist circumferences ranged between 61 and 120 cm with a mean of 90.86 ± 11.94. Regarding deformities, they were present in 68 (60.71%) patients while rheumatoid nodules were present also in 18 (16.07%) patients. At the time of the study, 10 (8.93%) patients only were not using NSAIDs. 58 patients (51.8%) were using steroids. Ninety patients (80.4%) were using antimalarial drugs, while 98 patients (87.5%) were using MTX. Twenty patients (17.9%) were using Sulphasalazine. 44 (39.3%) patients were using Leflunomide.

Table 2 reported the comparison of the laboratory parameters in patient and control groups.

There were highly statistical significant difference between patients and controls as regarding the prevalence of subclinical atherosclerosis; thirty-five patients (31.3%) have thickened IMT and only 6 patients (5.4%) had atherosclerotic plaque; meanwhile, only two (5%) of controls had thickened intima with no detected plaque formation.

Table 3 summarized the anthropometric features, traditional cardiovascular risk factors, and disease features in two groups (patients with atherosclerosis and patients without) divided according to the IMT more than 0.72 mm with IMT equal to or less than 0.72 mm. A significant difference was detected in both duration of the disease (*P* < 0.000^***^) and patient's age (*P* < 0.01^*^). There is a significant association between the cumulative doses of steroids and the thickened IMT (*P* < 0.005^**^). Also there is significant difference in Us-CRP (*P* < 0.000^***^), DAS28 (*P* < 0.002^**^). We must notice that cut-off value of IMT was different in different studies; some studies showed carotid artery IMT ≥0.60 mm was a marker of atherosclerosis; if we take this threshold in our mind, we found the same number of our patient had atherosclerosis. But another author showed carotid artery IMT >0.90 mm was a marker of atherosclerosis; the number of our patients who had IMT >0.90 mm was only four (3.6%).

**Figure 1:**
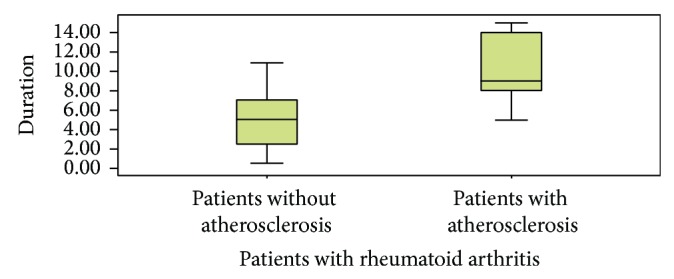
Comparison between patients with and without atherosclerosis with regard to disease duration.

**Figure 2:**
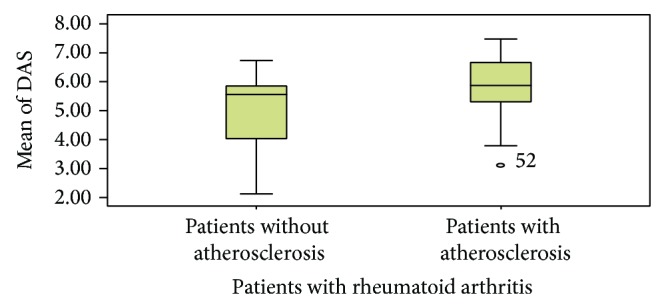
Comparison between patients with and without atherosclerosis with regard to disease activity.

The mean and standard deviation of disease duration of all patients was 6.4 ± 3.9. The mean of disease duration in patients with and without atherosclerosis was 10.5 ± 3.1 and 4.7 ± 2.9, respectively. The mean of disease activity index in patients was 5.2 ± 1.68 and the mean of disease activity index in patients with and without atherosclerosis was 5.8 ± 1.5 and 2.8 ± 1.3, respectively (Figures 1 and 2). There is highly statistically significant relation between presence of atherosclerosis with disease duration and disease activity (*P* < 0.0001^***^, *P* < 0.002^**^, resp.).

*Intimal Medial Thickness*. Tables 4, 5, and 6 showed a comparison of ultrasonographic duplex findings in RA patients and its subgroups with controls. The comparison between group 1 and group 11 and comparison between group 1A and group 11 with respect to ultrasonographic duplex findings of carotid arteries showed the mean maximum and left IMT were significantly higher in patients compared to the controls, while the comparison between group 1B and group 11 reported no significant differences.

Table 7 summarized the comparison of ultrasonographic duplex findings of carotid arteries in RA patients; the mean, maximum, and left IMT were also significantly higher in group 1A.

*Endothelial Function*. Tables 8, 9, and 10 showed a comparison of parameters which asses the endothelial function between all patients, patients subgroups with controls, and reported that post FMD (*P* < 0.000^***^), FMD dilatation percent (*P* < 0.000^***^), and dilatation ratio (*P* < 0.000^***^) were significantly lower in patient than controls, even in group 1B.

Table 11 showed a comparison of parameters which asses the endothelial function in RA patients groups; post FMD (*P* < 0.04^*^), FMD dilatation percent (*P* < 0.03^*^), and dilatation ratio (*P* < 0.01^*^) were significantly lower in the first group than the second.

*Atherosclerosis and Steroid Use.* There are 58 patients (51.8%) of current steroid users and 54 patients (48.2%) are nonsteroid users in RA patients, nearly similar prevalence of cases with increased mean IMT in patient using glucocorticoids (18 cases; 51.4% of the thickened IMT) than nonusers (17 cases, 48.6% of the thickened IMT). No significant differences were found in comparison of parameters which asses the endothelial function in RA patients and ultrasonographic duplex of their carotid arteries in patients who used steroids with patients who do not use steroids (Tables 12, 13, and 14).

*Atherosclerosis and Disease Activity.* There is association between DAS28 and IMT and presence of plaques with a significant negative correlation between DAS28 score and endothelial function parameters in RA patients, FMD dilatation percent and dilatation ratio (*P* < 0.005^**^, *P* < 0.007^**^, resp.), and also it has a significant correlation with ultrasonographic duplex findings of carotid arteries maximum, left IMT, and mean IMT (*P* < 0.01^*^, *P* < 0.001^**^, and *P* < 0.0001^***^, resp.) (Tables 15 and 16).

## 4. Discussion

In the present study there is an overall higher prevalence of premature atherosclerosis in RA patients than controls; in addition, the mean IMT was significantly higher in RA patients than our healthy controls (0.57 ± 0.051 versus 0.49 ± 0.072 mm, resp.) (*P* = 0.01^*^). In addition to the two markers of subclinical atherosclerosis; the mean right and left IMT were higher in patients compared to the controls. Plaques were also more frequently observed in RA (*P* = 0.02). And by comparison of parameters which asses the endothelial function in RA patients and controls. Only FMD (*P* = 0.043) and dilatation ratio (*P* = 0.001) were significantly lower in patient than controls. These result in agreement of other studies [20–22].

In accordance with our result, a study by La Montagna and his colleague [21] confirmed that the prevalence of the atherosclerosis is higher in RA patients than in the general population.

Our finding indicated that accelerated atherosclerosis is a well-defined feature of RA which was in agreement with many other studies [23–25].

In our study, the cut-off point of intima media thickness is considered abnormal if  >0.072 cm which is in agreement with other studies [21, 26, 27]. The cut-off point between normal and high IMT was different between studies. In Doria and his colleagues [13] study normal IMT was defined when complex intima media is <0.09 cm; therefore, IMT values 0.09 cm were considered indicative of thickened intima. While in Marasini et al. [14] study, the subclinical atherosclerosis IMT cut-off value detected was >0.7 mm. Differences in the ultrasound equipment or even using the same equipment with different frequency or different resolution could result in such variability. Common carotid IMT using ultrasound with a high frequency may allow more accurate results than of limited resolution. Different scanning and reading protocols could also influence the results. Another factor reported that IMT in men is significantly thicker than that in women when estimated using 7-8 MHz frequency ultrasound [28], difference in the methodology for assessment of atherosclerosis may lead to different results.

Disease duration is the best predictor of plaque and atherosclerosis development. We did not find evidence of this in patients with short disease duration (the mean and standard deviation was 10.5 ± 3.1). This was in agreement with other authors [29] who showed no atherosclerosis in disease duration <7 years.

In our study, there was no significant difference found between the two groups.

As regarded the prevalence of traditional cardiovascular risk factors. In our study, obesity, as defined by BMI >30.0, was reported in 40 (35.7%) of our RA patients versus 12 (30%) of our healthy controls with no significant statistical differences between both groups. Our results were similar to other study [21]. Most authors have pointed out low levels of HDL-cholesterol [30, 31]. However, we did not find this association in the present study, this agrees with results of other studies [21]. The discrepancy in results is likely to depend on differences among the cohorts of patients investigated, including differences in their nutritional habits. Nevertheless traditional risk factors clearly play a role in the pathogenesis of atherosclerosis in RA and must be investigated when evaluating the individual RA patient [32].

Inflammation is considered to be an important risk factor for premature atherosclerosis in RA. This was a very consistent finding in the present study as measured by acute phase reactants including high level erythrocyte sedimentation rate and CRP. As expected, higher levels of ESR (*P* < 0.001) were detected in RA compared to healthy controls; this is in agreement with the previous study [22]. These data definitely support systemic inflammation as a factor of importance for premature atherosclerosis in RA patients.

In this study, the relationship with DAS28, a composite disease activity index in RA, supports in role for chronic inflammation in the development of atherosclerosis. The relationship between IMT, DAS28, and glucocorticoid cumulative doses confirmed that atherosclerosis in RA is associated with inflammation in agreement with La Montagna and his colleague [21].

In this study the percentages of steroid user are 51.8% and nonusers are 48.2% in RA patients. No significant differences were found between the two groups in ultrasonographic duplex of their carotid arteries; and no significant differences were found in the endothelial function; but there is a significant association between the cumulative doses of steroids and the thickened IMT (*P* < 0.05^*^). Other authors saw that patients requiring GCs as part of their disease management are likely to have aggressive disease, which contributes to a reduction in mobility as a result of joint stiffness and damage. Therefore, GC use in this subset of patients may result in suppression of disease activity and allow patients to lead a more active lifestyle with a further beneficial effect on HDL levels as well as aggressive control of inflammatory disease activity with available means, including judicious usage of GCs [33]. Thus, accurate drug history and accurate dose determination was a problem, exclusively in illiterate patients and on those with long disease duration. In practice, therefore, judicious use of steroids to control inflammation is probably beneficial. Excessive dosing may exacerbate metabolic factors. Achieving the correct balance, therefore, remains a major clinical challenge.

## 5. Conclusion

Egyptian asymptomatic RA patients exhibited increased carotid IMT and impaired FMD with increasing the frequency of atherosclerosis compared with general population. Thus carotid ultrasonography and endothelial function by flow mediated vasodilatation must be done in all RA patients, which could be simple noninvasive method of identifying preclinical atherosclerosis, in addition to the need for control of rheumatoid disease activity and its inflammatory burden because uncontrolled chronic inflammation is probably the major factor for premature atherosclerosis.

## Figures and Tables

**Table 1:** Demographic data of patients and control group.

		Patients (*N* = 112)	Control (*N* = 40)	*P* value
Age	Range	21–49	22–49	0.350
	Mean ± St.D.	36.50 ± 7.22	34.6 ± 8.22	

Sex	Male	18 (16.07%)	6 (15.8%)	0.977
	Female	94 (83.92%)	32 (84.2%)	

**Table 2:** Laboratory features of RA patients and controls.

	RA patients (*n* = 112)	Controls (*n* = 40)	*P*
	Mean ± SD	Mean ± SD	
Hb (gm%)	11.82 ± 2.04	12.82 ± 1.79	0.05
WBCs (/mm)	6.08 ± 2.28	7.46 ± 2.74	0.5
Platelets (/mm)	286.68 ± 88.33	269.79 ± 60.95	0.4
ESR (mm/hr)	**40.82 ± 42.27**	** 7.74 ± 1.3**	0.000^***^
FBS (mg%)	90.7 ± 17.43	88.42 ± 10.27	0.5
AlT	23.05 ± 11.61	21.37 ± 8.32	0.5
AsT	24.73 ± 21.14	24.37 ± 8.48	0.9
Total cholesterol (mg/dL)	166.25 ± 30.76	170.42 ± 36.65	0.63
Triglycerides (mg/dL)	90.70 ± 30.72	81.42 ± 26.8	0.22
HDL (mg/dL)	42.8 ± 3.4	42.37 ± 4.4	0.63
LDL (mg/dL)	114.45 ± 33.48	122.26 ± 35.41	0.406

Hb = hemoglobin, WBC = white blood cells, ESR = erythrocyte sedimentation rate, FBS = fasting blood sugar, AlT = alanine transaminase, AsT = aspartate transaminase, and RF = rheumatoid factor.

^***^Highly significant.

**Table 3:** Comparison of patients features between patients with and without atherosclerosis.

	Patients without atherosclerosis (*n* = 77)	Patients with atherosclerosis (*n* = 35)	*P*
	Mean ± SD	Mean ± SD	
Age	34.9 ± 6.9	40.12 ± 6.5	0.01^*^
Disease duration	4.7 ± 2.9	10.5 ± 3.1	0.000^***^
DAS 28	2.85 ± 1.35	5.8 ± 1.5	0.002^**^
Obesity (BMI)	26.7 ± 5.4	24.5 ± 8.2	0.23
Systolic BP (mm/Hg)	117.23 ± 8.13	120.29 ± 10.38	0.37
Diastolic BP	76.81 ± 4.83	78.82 ± 5.16	0.21
Us-CRP	48.48 ± 51.599	120.79 ± 53.89	0.0001^***^
Rheumatoid factor	87 ± **21.1**	235.9 ± **37.8**	0.5
Total cholesterol (mg/dL)	162.15 ± 28.6	175.7 ± 34.4	0.13
Triglycerides (mg/dL)	87.1 ± 30.6	98.8 ± 30.15	0.21
HDL (mg/dL)	43.33 ± 4.4	42.85 ± 3.3	0.07
LDL (mg/dL)	110.62 ± 31.45	123.24 ± 37.2	0.2
Cumulative steroid	748.21 ± 942.575	1335.88 ± 1621.50	0.005^**^
HAQ	0.48 ± 0.09	0.49 ± 0.12	0.3
SENS	11.8 ± 1.9	13.4 ± 3.2	0.9

DAS 28 = disease activity score, BMI = body mass index, BP = blood pressure, Us-CRP = ultra sensitive c-reactive protein, IR = insulin resistance, HDL = high density lipoprotein, LDL = low density lipoprotein, HAQ = health assessment questionnaire. SENS = Simple Erosion Narrowing Score. ^**^Moderate significant and ^***^highly significant.

**Table 4:** Comparison of ultrasonographic duplex findings in RA patients and controls.

	RA patients (*n* = 112)	Controls (*n* = 40)	*P*
	Mean ± SD	Mean ± SD	
Mean IMT	0.057 ± 0.016	0.050 ± 0.007	0.018^*^
Left IMT	0.057 ± 0.016	0.049 ± 0.008	0.01^*^
Right IMT	0.068 ± 0.087	0.0495 ± 0.0869	0.11
Maximum	0.0597 ± 0.017	0.0526 ± 0.008	0.02^*^

IMT = intima media thickness.

^*^Significant.

**Table 5:** Comparison of ultrasonographic duplex findings in group 1A patients and controls.

	Group 1A (*n* = 92)	Controls (*n* = 40)	*P*
	Mean ± SD	Mean ± SD	
Mean IMT	0.058 ± 0.016	0.050 ± 0.007	0.01^*^
Left IMT	0.0579 ± 0.01	0.049 ± 0.008	0.01^*^
Right IMT	0.052 ± 0.096	0.0495 ± 0.0869	0.1
Maximum	0.059 ± 0.017	0.0526 ± 0.008	0.01^*^

IMT = intima media thickness.

^*^Significant.

**Table 6:** Comparison of ultrasonographic duplex findings between group 1B and controls.

	Group 1B (*n* = 20)	Controls (*n* = 40)	*P*
	Mean ± SD	Mean ± SD	
Mean IMT	0.054 ± 0.017	0.050 ± 0.007	0.4
Left IMT	0.054 ± 0.017	0.049 ± 0.008	0.4
Right IMT	0.053 ± 0.017	0.0495 ± 0.0869	0.4
Maximum	0.056 ± 0.018	0.0526 ± 0.008	0.6

IMT = intima media thickness.

**Table 7:** Comparison of ultrasonographic duplex findings in RA patients.

	Group 1A (*n* = 92)	Group 1B (*n* = 20)	*P*
	Mean ± SD	Mean ± SD	
Mean IMT	0.057 ± 0.011	0.053 ± 0.017	0.04^*^
Lt. IMT	0.058 ± 0.016	0.054 ± 0.017	0.01^*^
Rt. IMT	0.052 ± 0.096	0.053 ± 0.017	0.55
Maximum	0.059 ± 0.017	0.056 ± 0.018	0.04^*^

IMT = intima media thickness.

^*^Significant.

**Table 8:** Comparison of endothelial function in RA patients and controls.

	RA patients (*n* = 112)	Controls (*n* = 40)	*P*
	Mean ± SD	Mean ± SD	
Pre. fmd (average)	3.69 ± 0.51	3.9 ± 0.49	0.121
Post. fmd (average)	4.32 ± 0.60	5.11 ± 0.52	0.000^***^
Fmd dilatation %	17.30 ± 7.72	28.52 ± 10.11	0.000^***^
Pre. GTN (average)	3.71 ± 0.54	3.91 ± 0.49	0.150
Post. GTN (average)	4.63 ± 0.72	4.89 ± 0.62	0.131
GTN dilatation %	24.87 ± 9.02	25.72 ± 9.67	0.727
Dilatation ratio	0.71 ± 0.261	1.078 ± 0.189	0.000^***^

FMD = flow mediated dilatation, GTN = glyceryl trinitrate.

^***^Highly significant.

**Table 9:** Comparison of endothelial function between group 1A patients and controls.

	Group 1A (*n* = 92)	Controls (*n* = 40)	*P*
	Mean ± SD	Mean ± SD	
Pre-FMD (average)	3.7 ± 0.52	3.9 ± 0.49	0.1
Post-FMD (average)	3.43 ± 0.6	5.109 ± 0.52	0.000^***^
FMD dilatation %	16.26 ± 7.35	28.52 ± 10.11	0.000^***^
Pre-GTN (average)	3.14 ± 0.56	3.91 ± 0.49	0.2
Post-GTN (average)	4.64 ± 0.71	4.89 ± 0.62	0.14
GTN dilatation %	24.33 ± 9.1	25.72 ± 9.67	0.5
Dilatation ratio	0.69 ± 0.25	1.078 ± 0.189	0.000^***^

FMD = flow mediated dilatation, GTN = glyceryl trinitrate.

^***^Highly significant.

**Table 10:** Comparison of endothelial function between group 1B and controls.

	Group 1B (*n* = 20)	Controls (*n* = 40)	*P*
	Mean ± SD	Mean ± SD	
Pre-FMD (average)	3.56 ± 0.46	3.9 ± 0.49	0.07
Post-FMD (average)	4.34 ± 0.7	5.109 ± 0.52	0.005^**^
FMD dilatation %	22.07 ± 8.8	28.52 ± 10.11	0.005^**^
Pre-GTN (average)	3.59 ± 0.46	3.91 ± 0.49	0.1
Post-GTN (average)	4.61 ± 0.81	4.89 ± 0.62	0.2
GTN dilatation %	27.32 ± 8.32	25.72 ± 9.67	0.6
Dilatation ratio	0.83 ± 0.29	1.078 ± 0.189	0.01^*^

FMD = flow mediated dilatation, GTN = glyceryl trinitrate.

^*^Significant. ^**^Moderate significant.

**Table 11:** Comparison of endothelial function in subdivisions of RA patients.

	Group 1A (*n* = 92)	Group 1B (*n* = 20)	*P*
	Mean ± SD	Mean ± SD	
Pre. fmd (average)	3.73 ± 0.52	3.56 ± 0.46	0.121
Post. fmd (average)	3.43 ± 0.59	4.34 ± 0.6	0.04^*^
Fmd dilatation %	16.26 ± 7.35	22.07 ± 8.8	0.03^*^
Pre. GTN (average)	3.14 ± 0.56	3.59 ± 0.46	0.4
Post. GTN (average)	4.64 ± 0.71	4.61 ± 0.81	0.9
GTN. dilatation %	24.33 ± 9.1	27.32 ± 8.32	0.3
Dilatation ratio	0.68 ± 0.25	0.83 ± 0.29	0.01^*^

FMD = flow mediated dilatation, GTN = glyceryl trinitrate.

^*^Significant.

**Table 12:** Percentages of steroid user to nonusers in RA patients and the relationship with thickened IMT.

RA patients *N* = 56	IMT ≤ 0.72 mm	IMT > 0.72 mm
Steroids user *n* = 58 (51.7%)	40	18

Steroids nonuser *n* = 54 (48.2%)	37	17

IMT = intima media thickness.

**Table 13:** Comparison of parameters which assess the endothelial function in RA patients who use steroids and RA patients who do not use steroids.

	RA patients who use steroids (*n* = 58)	RA patients who do not use steroids (*n* = 54)	*P*
	Mean ± SD	Mean ± SD	
Pre-FMD (average)	3.67 ± 0.56	3.73 ± 0.45	0.6
Post-FMD (average)	4.33 ± 0.61	4.3 ± 0.6	0.86
FMD dilatation %	18.31 ± 7.78	15.86 ± 7.93	0.2
Pre-GTN (average)	3.7 ± 0.59	3.75 ± 0.47	0.6
Post-GTN (average)	4.63 ± 0.74	4.6 ± 0.7	0.95
GTN dilatation %	26.03 ± 8.01	23.19 ± 10.3	0.3
Dilatation ratio	0.7 ± 0.26	0.72 ± 0.26	0.8

FMD = flow mediated dilatation, GTN = glyceryl trinitrate.

**Table 14:** Comparison of ultrasonographic duplex findings in RA patients who use steroids and RA patients who do not use steroids.

	RA patients who use steroids (*n* = 58)	RA patients who do not use steroids (*n* = 54)	*P*
	Mean ± SD	Mean ± SD	
Mean IMT	0.058 ± 0.016	0.055 ± 0.016	0.53
Left IMT	0.059 ± 0.017	0.055 ± 0.015	0.54
Right IMT	0.078 ± 0.12	0.055 ± 0.017	0.3
Maximum	0.061 ± 0.018	0.058 ± 0.016	0.5

IMT = intima media thickness.

**Table 15:** Correlation between DAS28 and endothelial function in RA patients.

Endothelial function	DAS28	
	(*r*)	*P*
Pre-FMD (average)	0.064	0.23
Post-FMD (average)	0.048	0.07
FMD dilatation %	−0.249	0.005^**^
Pre-GTN (average)	0.044	0.75
Post-GTN (average)	0.028	0.79
GTN dilatation %	0.002	0.9
Dilatation ratio	−0.355	0.007^**^

(*r*) = Correlation coefficient, FMD = flow mediated dilatation, and GTN = glyceryl trinitrate.

^**^Moderate significant.

**Table 16:** Correlation between DAS 28 and ultrasonographic duplex findings in RA patients.

Duplex findings	DAS 28	
	(*r*)	*P*
Right IMT	0.110	0.42
Left IMT	0.339	0.001^**^
Mean IMT	0.324	0.000^***^
Maximum	0.321	0.01^*^

IMT = intima media thickness, (*r*) = correlation coefficient.

^*^Significant, ^**^moderate significant, and ^***^highly significant.

## Recommendation

Research focusing on sensitive predictors of premature atherosclerosis including biomarkers like adhesive molecule, proinflammatory cytokines, and chemokines should be addressed so that some intervention model could be prepared for the prevention of cardiovascular-disease-related morbidities and mortalities in the RA population.
